# A Multisite Investigation of Areas for Improvement in COVID-19 Surge Capacity Management

**DOI:** 10.1089/hs.2023.0019

**Published:** 2023-09-26

**Authors:** Emily R. Post, Reena Sethi, Adeteju A. Adeniji, Clark J. Lee, Sophia Shea, Rebecca Metcalf, Jamie Gaynes, Kila Tripp, Thomas D. Kirsch

**Affiliations:** Emily R. Post, PhD, is a Research Associate, at The Henry M. Jackson Foundation for the Advancement of Military Medicine, Inc., Bethesda, MD, supporting The National Center for Disaster Medicine and Public Health, Uniformed Services University of the Health Sciences, Bethesda, MD.; Reena Sethi, DrPH, MHS, is a Senior Public Health Lead Researcher, at The Henry M. Jackson Foundation for the Advancement of Military Medicine, Inc., Bethesda, MD, supporting The National Center for Disaster Medicine and Public Health, Uniformed Services University of the Health Sciences, Bethesda, MD.; Adeteju A. Adeniji, MPH, is a Research Project Administrator, at The Henry M. Jackson Foundation for the Advancement of Military Medicine, Inc., Bethesda, MD, supporting The National Center for Disaster Medicine and Public Health, Uniformed Services University of the Health Sciences, Bethesda, MD.; Clark J. Lee, JD, MPH, is a Research Associate, at The Henry M. Jackson Foundation for the Advancement of Military Medicine, Inc., Bethesda, MD, supporting The National Center for Disaster Medicine and Public Health, Uniformed Services University of the Health Sciences, Bethesda, MD.; Sophia Shea, MPH, is a Project Manager, Global Center for Health Security, University of Nebraska Medical Center, Omaha, NE.; Rebecca Metcalf, MPP, is a Senior Manager, Deloitte Consulting LPP, Arlington, VA.; Kila Tripp is a Consultant, Deloitte Consulting LPP, Arlington, VA.; Jamie Gaynes, MPH, is a Manager, Deloitte Consulting LPP, Boston, MA.; Thomas D. Kirsch, MD, MPH, FACEP, was Director (Retired), at The Henry M. Jackson Foundation for the Advancement of Military Medicine, Inc., Bethesda, MD, supporting The National Center for Disaster Medicine and Public Health, Uniformed Services University of the Health Sciences, Bethesda, MD.

**Keywords:** National Disaster Medical System (NDMS), COVID-19, Surge capacity, Surge event, Definitive care, Emergency preparedness

## Abstract

The congressionally authorized National Disaster Medical System Pilot Program was created in December 2019 to strengthen the medical surge capability, capacity, and interoperability of affiliated healthcare facilities in 5 regions across the United States. The COVID-19 pandemic provided an unprecedented opportunity to learn how participating healthcare facilities handled medical surge events during an active public health emergency. We applied a modified version of the Barbisch and Koenig 4-S framework (*staff, stuff, space, systems*) to analyze COVID-19 surge management practices implemented by healthcare stakeholders at 5 pilot sites. In total, 32 notable practices were identified to increase surge capacity during the COVID-19 pandemic that have potential applications for other healthcare facilities. We found that *systems* was the most prevalent domain of surge capacity among the identified practices. *Systems* and *staff* were discussed across all 5 pilot sites and were the 2 domains co-occurring most often within each surge management practice. These results can inform strategies for scaling up and optimizing medical surge capability, capacity, and interoperability of healthcare facilities nationwide. This study also specifies areas of surge capacity worthy of strategic focus in the pilot's planning and implementation efforts while more broadly informing the US healthcare system's response to future large-scale, medical surge events.

## Introduction

Ahealthcare facility's surge capacity is its ability to manage and expand care capabilities in response to a sudden increase in patient volume that exceeds typical daily operations.^1,2^ Surge capacity is thus a critical component of effective healthcare provision in response to disasters or conflicts that produce high casualty counts.^3^ The ongoing COVID-19 pandemic has demonstrated how limited surge capacity can lead to increased patient morbidity,^4^ disrupt economic functioning,^5^ and exacerbate structural and social inequities that delay recovery from the original disaster event.^6^ These circumstances suggest that increasing surge capacity has the potential to not only save thousands of lives but also ensure the security and ongoing stability of the nation's healthcare system, economy, and governing institutions in the aftermath of crises.

The National Disaster Medical System (NDMS) was created in 1984 with the objective of providing a unified medical system to effectively transport and care for combat casualties resulting from a large-scale overseas conflict, as well as to supplement state and local medical resources during domestic emergencies.^7-9^ Today, the US federal government primarily uses the NDMS to respond to increased healthcare needs during local and regional disasters by deploying disaster medical assistance teams.^10,11^ The definitive care component of the NDMS is rarely used and has never been activated to care for military casualties during a war.^8,10^ And yet, the NDMS has grown to include a national network of over 1,700 healthcare facilities that agree to provide definitive medical care in the event of a national disaster or public health emergency.^10,11^ It is currently unknown whether the existing NDMS system can provide the scale and scope of definitive medical care necessary to meet the health security challenges of the 21st century.

In response to this potential threat, Congress authorized and directed the US Department of Defense to conduct an NDMS Pilot Program in the National Defense Authorization Act for Fiscal Years 2020 and 2021.^12,13^ The pilot aims to improve the medical surge capability and capacity of the definitive care component of the NDMS over 5 years. Five geographic sites comprising coordinated regional networks of federal and civilian partners were selected as testing grounds for assessing and implementing plans to coordinate healthcare response during a large-scale overseas conflict.^14^ Site locations include the National Capital Region/Metropolitan Washington, DC; Omaha, Nebraska; Denver, Colorado; San Antonio, Texas; and Sacramento, California. The pilot's launch in early 2020 coincided with the emergence of the SARS-CoV-2 virus.

The COVID-19 pandemic demonstrated that many US hospitals faced significant challenges managing surge capacity.^15,16^ This situation provided concrete examples of how pilot site facilities responded to an actual surge event involving a substantial surge of patients. Pandemic surges forced healthcare and public health leaders to constantly adapt to changing conditions with limited time, preparation, or resources, which led to a period of rapid innovation in the nation's healthcare system. By necessity, new practices for managing capacity surges were designed, tested, and implemented at each pilot site. To make the most of this opportunity, the pilot identified novel strategies that NDMS-partnering healthcare facilities put into practice to effectively expand and manage surge capacity during the initial 2 years of the COVID-19 pandemic. This analysis functioned to inform short-term planning for the pilot and long-term planning to improve the NDMS definitive care network.

Our study reports on this collection of surge management practices implemented at pilot site healthcare facilities during the COVID-19 pandemic and then systematically analyzes the data based on the 4-S framework of surge capacity developed by Barbisch and Koenig.^17^ This framework organizes surge capacity into 4 distinct domains: *staff* (appropriately trained personnel), *stuff* (durable and perishable supplies and equipment), *structures* (physical facilities and structures where healthcare happens), and *systems* (management policies and procedures that integrate operations between different entities within a healthcare organization, or between a healthcare organization and external supporting entities). The framework was developed to capture the multifaceted nature of this concept, and practitioners argue that surge capacity is best achieved when these essential components are seamlessly integrated into a healthcare system.^17,18^

Through our secondary analysis of previously collected data, we determined which of the 4 domains appeared most prevalently among the identified surge management practices and, of these, which domain(s) appeared most prevalently across the 5 pilot sites. The NDMS definitive care network can integrate more precise improvements into future surge event response if its component healthcare facilities can identify consequential practices that are noted as important for improving medical surge operations and can pinpoint the primary domain of surge capacity that typifies these practices. Findings from this study can be applied nationally to other healthcare facilities seeking guidance on innovations and adjustments to expand their facility's surge capacity and capabilities.

## Methods

### Data Source

COVID-19 surge management practices were initially identified through guided discussion forums hosted by pilot team personnel at each of the 5 sites, involving a total of 342 participants, between January and March 2022 (Table 1). Each of the 5 forums involved a series of multiday sessions where group discussions were conducted among a selection of healthcare, public health, and emergency management stakeholders local to the specific pilot site. Participants were chosen based on purposive sampling methods that prioritized high-level expertise in healthcare system management and practical knowledge in handling COVID-19 surge events. Moderators of discussion forums used facilitator guides to direct conversations on which medical surge innovations were considered important to healthcare facilities during the COVID-19 pandemic (Supplemental material: Appendix A; www.liebertpub.com/doi/suppl/10.1089/hs.2023.0019). Data were restricted to surge management practices implemented during the first 2 full years of the COVID-19 pandemic, from March 1, 2020, to March 31, 2022. Discussion comments that articulated strategies for responding to surge capacity were tracked through detailed notetaking.

**Table 1.** Descriptive Statistics of Primary Data Contributions by NDMS Pilot Site

NDMS Pilot Program Site	Number of Discussion Forum Participants	Number of Associated Surge Management Practices
NCR	85	6
Omaha, NE	106	10
Denver, CO	54	7
San Antonio, TX	30	13
Sacramento, CA	67	4
Totals	342	40

Note: In some instances, the same surge management practice was mentioned at multiple pilot sites. As a result, the combined number of surge management practices associated with each pilot site (n=40) is greater than the number of individual practices identified in this study (n=32). Abbreviations: NCR, National Capital Region/Metropolitan Washington, DC; NDMS, National Disaster Medical System.

Primary data from these discussion forums were initially reviewed by pilot personnel. Group discussions were then conducted via Zoom with a smaller selection of participants who had attended the multiday group discussion to refine the selection of practices discussed in each corresponding forum. Participants were again chosen by purposive sampling that prioritized key local and regional leaders of the 5 pilot sites. Discussion moderators used facilitator guides to direct conversations on reviewing the initial list of identified notable practices and provide insight into the validity and core concepts of each (Supplemental material: Appendix A). This process produced the finalized list of notable practices in managing surge capacity.

### Data Analysis

Using a deidentified version of the aforementioned program planning data, 3 pilot-affiliated researchers separately coded surge management practices according to a popularly modified version of the 4-S framework that replaces *structures* with the term *space* to highlight the physical nature of healthcare facilities required to respond to surge.^3,17^ Definitions of each surge capacity domain were developed deductively from the scientific literature on surge capacity conceptual framing (Supplemental material: Appendix B). Each surge management practice was categorized based on the domain specified to be the most critical to the innovation's function, referred to as its *primary surge capacity domain*. When applicable, practices with overlapping functions were organized by a secondary surge capacity domain. The 3 coders then compared data and provided supporting arguments for each coding decision. Divergent coding was resolved by reviewing operational definitions of each surge capacity domain and reaching majority consensus on the appropriate coding decision.

### Research Ethics Review

The Human Research Protections Program Office for the Uniformed Services University of the Health Sciences determined that the protocol for this secondary analysis of previously collected deidentified data constituted research not involving human subjects and therefore did not require institutional review board review under relevant federal regulations and policy guidance on the protection of human research subjects.

## Results

The discussion forums produced a list of 32 notable surge management practices implemented at pilot site healthcare facilities to manage COVID-19 surge events (Table 2). *Systems* was the most prevalent domain of surge capacity, with more than half (n=21, 66%) of the identified surge management practices primarily concerning innovations to healthcare systems (Table 3). Furthermore, *systems* and *staff* were the only surge capacity domains referenced in at least 1 notable practice identified at all 5 pilot sites. Cross-tabulations of primary and secondary surge capacity domains also identified *systems* and *staff* as the domains most likely to co-occur within a single practice (Table 4).

**Table 2.** COVID-19 Surge Management Practices Organized by Associated Primary and Secondary Surge Capacity Domains

	COVID-19 Surge Management Practice	Primary Surge Capacity Domain	Secondary Surge Capacity Domain
1	Convene hospital/health system transfer center leads to coordinate patient transfers across the region using a hub-and-spoke model	Systems	Staff
2	Pair rural hospitals/health systems with urban hospitals to facilitate transfers in support of the hub-and-spoke model	Systems	Space
3	Coordinate staffing across the region to alleviate staffing shortages, particularly among postacute facilities that struggle to attract travel nurses	Staff	Systems
4	Identify vetted vendors of PPE and other supplies at a regional level to reduce the administrative research and sourcing burden on individual hospitals	Stuff	Systems
5	Leverage social media feedback to monitor and mitigate COVID-19 misinformation	Systems	–
6	Engage experienced “on-the-ground” personnel to train and share knowledge of learned best practices with new personnel	Staff	–
7	Temporarily suspend or modify regulations regarding state ambulance licensing	Systems	–
8	Temporarily extend licenses and certificates for EMS and emergency medical technician personnel	Staff	Systems
9	Engage local subject matter experts at the incident onset to support response operations	Staff	Systems
10	Centralize data to monitor hospital capacity across the state to inform patient movement	Systems	Space
11	Establish targeted workgroups as an avenue for dedicated conversation and collaboration on urgent issues	Systems	Staff
12	Include various types of organizations in hospital unified command groups and response efforts to create a comprehensive response and unified front	Systems	–
13	Utilize civilian patient placement capabilities (single-point transfer center)	Space	Systems
14	Modify surge plans to expand staffing and retain workforce	Staff	Systems
15	Routinely provide situational awareness between state and local agencies	Systems	
16	Create technology and process innovations to streamline resource-sharing during a medical surge response	Systems	Stuff
17	Pre-position memorandums of agreement between local hospitals and state government and local EMS and state governments concerning the use of state/local emergency management resources	Systems	Stuff
18	Centralize surge resource visibility within a single system to share staff more seamlessly across local healthcare organizations	Systems	Staff
19	Implement a centralized hotline to manage medical response-related services to alleviate administrative burdens	Systems	Staff
20	Bring chief executive officers from health systems across the region to expedite decisionmaking regarding patient movement	Systems	–
21	Establish strong existing interoperability between public health departments, EMS, and hospital systems to share the burden of response efforts during medical surge events	Systems	–
22	Use existing supply caches from previous emergencies and establish an ordering/delivery process	Stuff	Systems
23	Develop and disseminate daily status reports that include urban and rural medical surge data to help inform hospitals' response	Systems	–
24	Lessen the administrative burden for hospitals during a crisis by “owning” the load balancing work for the region	Systems	Staff
25	Establish crisis standards of care for healthcare staffing and EMS to ease the burden of managing an influx of patients	Systems	Staff
26	Track general trends in PPE needs and usage across the region to inform demand and supply	Stuff	Systems
27	Create shared data and dashboards across health systems and regions to inform hospital decisionmaking	Systems	–
28	Centralize information management and communication across health systems to create a unified front to the public and to reduce confusion	Systems	–
29	Collect critical data across health systems/organizations and store it in a central location	Systems	–
30	Leverage existing nonclinical staff to assist with administrative and operational activities related to surge management	Staff	–
31	Create a centralized operations center to oversee a region's response efforts to medical surge events	Systems	–
32	Include healthcare decisionmakers in workgroups and planning efforts	Staff	Systems

Abbreviations**:** EMS, emergency medical services; PPE, personal protective equipment.

**Table 3.** Descriptive Statistics of Surge Management Practices Categorized by the 4-S Framework of Surge Capacity

Primary Surge Capacity Domain	Number of Associated Practices	Number of Associated NDMS Pilot Program Sites	Associated NDMS Pilot Program Sites	Example Surge Management Practice
Space	1	1	Sacramento	Use civilian patient placement capabilities (ie, single-point transfer center)
Staff	7	5	NCR, Omaha, Denver, San Antonio, Sacramento	Coordinate staffing across regions to alleviate personnel shortages, particularly among postacute care facilities that struggle to attract travel nurses
Stuff	3	3	Omaha, Denver, San Antonio	Identify vetted vendors of PPE and other supplies at a regional level to reduce the administrative research and sourcing burden on individual hospitals
Systems	21	5	NCR, Omaha, Denver, San Antonio, Sacramento	Include various types of organizations in hospital unified command groups and response efforts to create a comprehensive response and unified front

Abbreviations: NCR, National Capital Region/Metropolitan Washington, DC; NDMS, National Disaster Medical System; PPE, personal protective equipment.

**Table 4.** Cross-Tabulation of Identified Surge Capacity Management Practices by Primary and Secondary Surge Capacity Domains

Primary Surge Capacity Domain	Secondary Surge Capacity Domain					
	Space	Staff	Stuff	Systems	None	Total
Space	0	0	0	1	0	1
Staff	0	0	0	5	2	7
Stuff	0	0	0	3	0	3
Systems	2	6	2	0	11	21
Total	2	6	2	9	13	32

## Discussion

This study identified 32 specific interventions that may be of value for healthcare preparedness planning and response during future medical surge events.

These notable practices ranged from detailed and highly specific (8: Temporarily extend licenses and certificates for emergency medical services and emergency medical technician personnel) to general and far-reaching (5: Leverage social media feedback to monitor and mitigate COVID-19 misinformation). A number of themes emerged when examining the collection of notable practices as a whole. Several explicitly mentioned patient transfer issues (1: Convene hospital/health system transfer center leads to coordinate patient transfers across the region using a hub-and-spoke model; 20: Bring chief executive officers from health systems across the region to expedite decisionmaking regarding patient movement). Another recurring topic was strategies for collecting and sharing information integral to hospital operations, such as monitoring patient care capacity (10: Centralize data to monitor hospital capacity across the state to inform patient movement) or staffing availability (18: Centralize surge resource visibility within a single system to share staff more seamlessly across local healthcare organizations). Several practices explicitly mentioned the creation of standardized information technology (IT) systems to centralize and easily disseminate this critical information (16: Create technology and process innovations to streamline resource-sharing during a medical surge response; 27: Create shared data and dashboards across health systems and regions to inform hospital decisionmaking). This follows calls, especially within NDMS hospitals, for increased integration between emergency management and IT systems, given its critical role in coordinating regional healthcare response during crises.^10^

This study then examined which of the 4 surge capacity domains was most prevalent among this collection of notable surge management practices implemented within pilot site facilities in response to the COVID-19 pandemic. *Systems* was the most prevalent domain of surge capacity observed among the analyzed practices. Additionally, the domains of *systems* and *staffing* were the only ones discussed at all 5 pilot sites and the domains most likely to co-occur within individual surge management practices. The emphasis on systems innovations to effectively manage surge capacity to COVID-19 response is best understood in context to the sustained scholarly debates about the centrality of *systems* to the concept of surge capacity.^3,18-20^ Many surge science experts consider *systems* to be the most essential element of the 4 as it ensures synchrony between the other domains.^2,18,21,22^ This viewpoint is supported by empirical studies tracking the efficacy of previous responses to surge events, which observe hospitals' foremost limiting factor to achieving surge capacity lying not in access to resources but in matching these resources to different organizational needs.^18,23^ Efficient resource-matching requires management policies and procedures (ie, *systems*) to effectively organize how staff, space, and stuff are used during a surge event. In essence, effective systems can be understood as the critical factor that allows surge capacity (command over resources) to be converted into surge capability (ability to apply resources for a desired outcome).^20^

The primacy of systems innovations among the observed surge management practices at pilot sites could reasonably be interpreted as underlining the importance of effective management and integration of the many internal and external components of healthcare necessary for adequate surge response. Several leading healthcare organizations have long advocated that healthcare facilities' surge capacity can be enhanced through cultivating cooperation with community organizations, and there has been a general trend in the past few decades championing the benefits of collaborating outside the traditional healthcare system.^2,24,25^ There have also been calls within the emergency planning community to establish management systems that more effectively support decisionmaking processes so that solutions can be made in a timely manner while inputs from personnel with relevant knowledge are appropriately consolidated.^26^ Several of these suggestions were mentioned in guided discussion forums as effective strategies for surge response within the 5 pilot sites. These observations may indicate empirical support to theoretical arguments for how impactful updates to healthcare facilities' systems can be for building surge capacity in response to medical surge events.

Alternatively, the heavy focus on systems innovations across pilot sites may also result in difficulties defining the concept. *Systems* is also the most conceptually broad domain in the 4-S framework, and while this article relies on one of the more common usages in the field, the term has various definitions in the literature. This is likely due to its less tangible nature, which cannot be as easily observed or measured compared with the finite resources of *staff, stuff*, and *space*. Depending on the source, procedures and policies falling under the *systems* domain can include legal precedents, financing rules, governance arrangements, bilateral and multilateral agreements, communications and transportation logistics, or procedural and organizational guidelines for how different staff collaborate during incidents.^3,20,27^ Previous scholars have suggested that to advance the field of surge capacity science, more precise analytical and conceptual frameworks are needed to determine which essential elements constitute an effective surge management system.^1^ In effect, the high prevalence of systems-focused practices in this study may also be interpreted as advancing the argument that greater conceptual clarity is needed on this topic.

*Systems* and *staff* were the domains most likely to co-occur among surge management practices analyzed in this study. Staffing issues are particularly common during surge events, as personnel are often unable, unwilling, or underprepared to carry out their full range of duties when a disaster impacts their community.^28-30^ This was especially true during the pandemic due to the impact of COVID-19 infections on the physical and mental health of healthcare staff, leading to an increase in short- and long-term attrition. Addressing increased patient loads during disasters and public health emergencies also often requires a complex rearrangement or redeployment of personnel.^31-34^ Systems that allow healthcare facilities to efficiently organize staff deployment based on training requirements, availability, and location have the obvious benefit of enabling them to be proactive in meeting patient needs in the face of uncertainty. These considerations may explain why these 2 surge capacity domains were paired so frequently in this study.

### Limitations

Both the timing and nonsystematic nature of primary data collection present limitations within this study. Surge management practices were identified during the ongoing COVID-19 pandemic, meaning that participants were actively responding to on-the-ground challenges related to local outbreaks (eg, early 2022 Omicron surge nationwide) and other natural hazards (eg, December 2021 Marshall Fire in Colorado) during the data collection process. This presented several challenges in gaining access to participants, necessitating a flexible approach to scheduling site-specific interviews with stakeholders. While this approach likely introduced bias into results, these methodological choices also efficiently provided access to context-rich data from subject matter experts with detailed insider knowledge of how the healthcare system at particular pilot sites responded to COVID-19.

The timing and nature of data collection also meant that many participating healthcare facilities had not yet been able to perform a comprehensive analysis or produce formal documentation (eg, after-action reports) tracking their experiences managing surge capacity and capabilities during the pandemic. As a result, those described do not necessarily represent an exhaustive list of all existing best practices that occurred at the participating healthcare facilities. This also meant that consistency in the level of detail provided for each identified practice varied across sites. It is likely that this inconsistency, combined with the variation in previous knowledge of the healthcare system between researchers who coded these practices, impacted levels of interrater reliability. While interrater reliability statistics are more likely to be artificially low despite high levels of coder agreement when handling qualitative data,^35^ an extension of this work could include increasing the number of coders and computing interrater agreement. Additionally, when the COVID-19 pandemic response eventually scales down, healthcare organizations will have the opportunity to more formally reflect on potential areas for improvement in managing surge capacity. Future research could productively expand on and provide additional support to the insights made in this article by analyzing a more detailed, systematically documented list of after-action reports and lessons learned produced by healthcare organizations in years to come.

Careful consideration should also be applied when assessing the generalizability of findings. While this study focused solely on NDMS healthcare facilities' ability to respond to a medical surge in response to a global pandemic, the original purpose of the NDMS is to handle surges of combat casualties returning from an overseas conflict, which pose their own specific medical planning challenges. Pandemics produce an influx of patients with similar illnesses and requiring similar critical care needs, whereas combat operations usually produce much wider variation in the types of illness or injuries presented, each with their own specific critical care needs, required facilities, and specialists^10^ (eg, burn units, vascular surgery, neurosurgical services). Potentially, systems that were appropriate for handling other types of medical surge events (eg, mass shootings, natural disasters, terrorist attacks) would not adequately translate to meeting the specific needs of a combat-casualty scenario.

Another factor impacting the generalizability of this study's results concerns the healthcare facilities included in the NDMS Pilot Program in relation to the overall US healthcare system. Each pilot site was selected partially due to its influential status in regional healthcare networks and location in populous metropolitan areas, making them healthcare hubs well-positioned to respond to state and national emergencies. This means each site played a critical “anchoring” role in their region's COVID-19 response. While this feature provided access to high-quality information from participants with real-world knowledge of emergency response, it also meant data was sourced from large, centrally located healthcare facilities in urban areas. These facilities operate at or near capacity nearly every day while also having more access to resources and support networks.^36,37^ It could be that surge management practices within these healthcare facilities prioritize different components of surge capacity than smaller or more rural facilities. Future empirical studies examining surge capacity innovations should include a more diverse selection of healthcare facilities and account for different types of surge events in their analyses.

## Conclusion

Our study identifies several suggested areas of improvement to increase surge capacity in response to the COVID-19 pandemic, the most significant public health emergency in recent memory. It also highlights the importance of systems-focused surge management practices in responding to the pandemic among healthcare facilities at NDMS Pilot Program sites. Due to the critical role systems play in organizing and enabling all aspects of the 4-S framework, strategically targeting improvements to this domain would likely have cascading effects on all other domains of surge capacity. This strategy can increase operational efficiency and program effectiveness within the pilot sites, throughout the NDMS definitive care network, and across the broader US healthcare system. Findings from this study can further expand current conversations in the healthcare preparedness and emergency management fields on how to best apply observations from the COVID-19 pandemic to bolster the stability and security of our nation's healthcare system. Despite contextual differences between a pandemic and other disaster and conflict scenarios, these medical surge capacity solutions can inform the planning, scaling, and response to other medical surge events.

## Supplementary Material

Supplemental data

Supplemental data

## Notes

[Supplementary-material SD1]

[Supplementary-material SD2]
