# Supplementary material for: A Multisite Investigation of Areas for Improvement in COVID-19 Surge Capacity Management
Source: Health Secur. 2023 Sep 26;21(5):333–40. doi: 10.1089/hs.2023.0019 (PMC10541923; doi:10.1089/hs.2023.0019)
Supplement: Supplemental data [file Suppl_AppendixA.docx]

**Supplemental Material**

Appendix A. Interview guides of questions distributed to study participants on notable COVID-19 surge-management practices. Group Discussion Forum Questions on Notable COVID-19 Surge Management Practices:

1. What examples of medical surge capability/capacity bright spots or innovations did you/your facility identify during the COVID-19 response?
2. What is/was a positive experience during the COVID-19 response that helped to improve public information sharing?
3. During COVID, did your organization share resources with external organizations/groups? If yes, what resources and with whom?
4. When COVID-19 hit, how helpful was your medical surge plan as written? Did you need to make amendments as COVID-19 unfolded? If yes, what amendments or changes did you make and why?
5. Does your organization have an AAR or other document summarizing bright spots you uncovered/implemented during the COVID-19 response? Would you be willing to share it with the FIT to inform long-range NDMS Pilot Program planning?
6. How can we improve medical surge planning based on COVID-19 response?
7. During the COVID-19 response, what data points have been used to measure medical surge capacity and/or capabilities during COVID? What were data points reported to measure medical surge capacity during COVID?

Validation of Notable COVID-19 Surge Management Practices:

1. Can you validate that the practices listed above are the correct leading practices?
2. Which practices are relevant to the NDMS wartime contingency scenario (WCS)?
3. Which leading practices are most critical to informing NDMS long-range planning?
4. Identify the leading practices that are exportable to other NDMS Pilot sites.
