# Supplementary material for: A Multisite Investigation of Areas for Improvement in COVID-19 Surge Capacity Management
Source: Health Secur. 2023 Sep 26;21(5):333–40. doi: 10.1089/hs.2023.0019 (PMC10541923; doi:10.1089/hs.2023.0019)
Supplement: Supplemental data [file Suppl_AppendixB.docx]

Appendix B. Operational definitions of surge capacity domains (Four-S Framework)

- *Staff:* Appropriately trained clinical, technical, and support personnel necessary to

provide healthcare services to patients and operate healthcare facilities

- *Stuff:* Durable and consumable supplies and operating equipment required to provide healthcare services to patients and support operations of healthcare facilities and staff
- *Space:* Physical facilities that contain the structural and functional capacity to provide healthcare services to patients
- *Systems:* Management policies, procedures, or operations that integrate healthcare facilities with specific departments within the organization, other healthcare facilities outside the organization, or larger public entities that must be coordinated and/or communicated with to provide healthcare services

References:

Barbisch DF, Koenig KL. Understanding Surge Capacity: Essential Elements. Acad Emerg Med. Nov 2006;13(11):1098-1102. doi:10.1197/j.aem.2006.06.041

Appendix B - Descriptive statistics of COVID-19 surge management practices by surge capacity domain and NDMS Pilot Program Site

| **Surge Capacity Domain** | **Number of Associated Practices -Primary** | **Number of Associated Practices -Secondary** | **Number of Associated Practices Total** | **Number of Associated Sites -Primary** | **Associated Sites -Primary** | **Number of Associated**  **Sites - Secondary** | **Associated Sites -Secondary** | **Number of Associated Sites Total** | **Associated Sites Total** |
| --- | --- | --- | --- | --- | --- | --- | --- | --- | --- |
| **Space** | 1 | 2 | 3 | 1 | Sacramento | 2 | Denver,  NCR | 3 | Denver, NCR, Sacramento |
| **Staff** | 7 | 7 | 14 | 5 | Denver,  NCR,  Omaha, Sacramento, San Antonio | 3 | Denver, Omaha,  San Antonio | 5 | Denver, NCR, Omaha, Sacramento, San Antonio |
| **Stuff** | 3 | 2 | 5 | 3 | Denver, Omaha,  San Antonio | 2 | Sacramento, San Antonio | 4 | Denver, Omaha, Sacramento, San Antonio |
| **Systems** | 21 | 9 | 30 | 5 | Denver,  NCR,  Omaha, Sacramento, San Antonio | 5 | Denver,  NCR,  Omaha, Sacramento, San Antonio | 5 | Denver, NCR, Omaha, Sacramento, San Antonio |
| **N/A** | NA | 13 | N.A. | N.A. | N.A. | N.A. | NA | NA | NA |
